# Exosomes released by *Brucella*‐infected macrophages inhibit the intracellular survival of *Brucella* by promoting the polarization of M1 macrophages

**DOI:** 10.1111/1751-7915.14274

**Published:** 2023-05-22

**Authors:** Yueli Wang, Honghuan Li, Zhenyu Xu, Jihai Yi, Wei Li, Chuang Meng, Huan Zhang, Xiaoyu Deng, Zhongchen Ma, Yong Wang, Chuangfu Chen

**Affiliations:** ^1^ College of Animal Science and Technology Shihezi University Shihezi China; ^2^ Xinjiang Center for Animal Disease Control and Prevention Urumqi China; ^3^ Jiangsu Key Laboratory of Zoonosis Yangzhou University Yangzhou China

## Abstract

Exosomes, membrane vesicles released extracellularly from cells, contain nucleic acids, proteins, lipids and other components, allowing the transfer of material information between cells. Recent studies reported the role of exosomes in pathogenic microbial infection and host immune mechanisms. *Brucella*‐invasive bodies can survive in host cells for a long time and cause chronic infection, which causes tissue damage. Whether exosomes are involved in host anti‐*Brucella* congenital immune responses has not been reported. Here, we extracted and identified exosomes secreted by *Brucella melitensis* M5 (Exo‐M5)‐infected macrophages, and performed in vivo and in vitro studies to examine the effects of exosomes carrying antigen on the polarization of macrophages and immune activation. Exo‐M5 promoted the polarization of M1 macrophages, which induced the significant secretion of M1 cytokines (tumour necrosis factor‐α and interferon‐γ) through NF‐κB signalling pathways and inhibited the secretion of M2 cytokines (IL‐10), thereby inhibiting the intracellular survival of *Brucella*. Exo‐M5 activated innate immunity and promoted the release of IgG2a antibodies that protected mice from *Brucella* infection and reduced the parasitaemia of *Brucella* in the spleen. Furthermore, Exo‐M5 contained *Brucella* antigen components, including Omp31 and OmpA. These results demonstrated that exosomes have an important role in immune responses against *Brucella*, which might help elucidate the mechanisms of host immunity against *Brucella* infection and aid the search for *Brucella* biomarkers and the development of new vaccine candidates.

## Graphical abstract

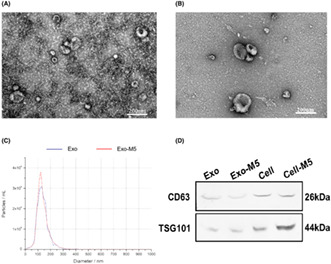

## INTRODUCTION

Brucellosis is a serious zoonosis caused by *Brucella* that results in abortion and infertility in several mammal species including humans (Yi et al., 2021). *Brucella* is a facultative intracellular bacteria with high selectivity and pathogenicity to its natural hosts. *Brucella* uses its surface virulence factors (lipopolysaccharides, outer membrane proteins and type IV secretion system) to parasitize macrophages for a long duration, resulting in persistent infection. Studies reported that exosomes that carry antigens and immunogenic factors during infection by pathogenic microorganisms might induce immune responses in hosts. Exosomes are tiny vesicles released extracellularly from cells. Intracellular pathogenic bacteria can survive in the host cell for a long time after invading the host and cause chronic infections, which can be harmful to the host (Zhao et al., 2020). At present, few studies have examined the relationship between exosomes and intracellular pathogenic bacteria, though it was shown that intracellular bacteria such as *Mycobacterium tuberculosis*, *Listeria monocytogenes* and *Salmonella* induced cells to secrete microvesicles (Garcia‐del Portillo et al., 1997). Other studies confirmed that microvesicles carrying *M. tuberculosis* antigens established protective immunity in mice (Giri & Schorey, 2008). Whether *Brucella* an intracellular bacteria – can induce macrophages to release exosomes requires further study.

Innate immunity is an important host defence mechanism that resists infection by pathogenic microorganisms. Macrophage polarization is a form of innate immune defence induced by external stimuli. Macrophages can be polarized into two subtypes: stimulation by interferon gamma (IFN‐γ) or lipopolysaccharides (LPS) leads to M1 macrophages phenotype, characterized by the production of nitric oxide synthase (iNOS) and inflammatory cytokines such as tumour necrosis factor alpha (TNF‐α) and interleukin‐6 (IL‐6). It mainly exhibits pro‐inflammatory activity and promotes the killing of pathogenic microorganisms by the host.

In contrast, the Th2 cytokines interleukin‐4 (IL‐4) and IL‐13 activate signal transducer and activator of transcription 6 (STAT6) to promote development of M2 macrophages phenotype. It secretes anti‐inflammatory cytokines such as IL‐4, IL‐10 and transforming growth factor‐β (TGF‐β) and high expression of arginase 1 (Arg1) and mannose receptor (CD206), which play important roles in tissue repair, allergic inflammation and helminth infection (Higuchi et al., 2016). Furthermore, M1 and M2 macrophages can be polarized to each other and their balance is important for cell homeostasis.

Exosomes carry material information between cells and participate in the activation of innate immunity (Wang et al., 2019). Studies reported that exosomes affect the biological function of macrophages by regulating their polarization, thereby affecting the activation of innate immunity. Other studies showed that exosomes mediated inflammatory responses of host cells through the NF‐κB pathway (Gao et al., 2016), which can induce macrophages to polarize to the M1 type, thereby indirectly promoting the release of Th1 type cytokines (Dai et al., 2020). In addition, innate immunity, as well as subsequent anti‐pathogenic effects, induced by exosomes mainly relies on the transmission of immunologically active substances between immune cells via exosomes. It has been reported that LPS‐pre‐exosome can improve the regulation of macrophage polarization and secretion of inflammatory cytokines through the Toll‐like receptor (TLR)4/TLR2/NF‐κB signalling pathway, thereby promoting NLRP3 inflammation to activate and enhance cell phagocytic ability (Ti et al., 2015). However, *Brucella* induces host innate immunity mostly in the early stages of infection, after which 90% of the bacteria are eliminated. Whether *Brucella* can induce the release of exosomes from macrophages that then induce macrophage polarization to affect the survival of bacterial cells requires further study.

This study examined how exosomes carrying *Brucella* antigens initiated innate immune responses in the early stages of *Brucella* infection, and investigated the effects of macrophage polarization induced by exosomes on the survival of *Brucella melitensis* M5 (*B. melitensis* M5) in cells. This will lay a theoretical foundation for the prevention and treatment of brucellosis and the development of new *Brucella* vaccine candidates.

## RESULTS

### Identification of exosomes by different methods

**FIGURE 1:**
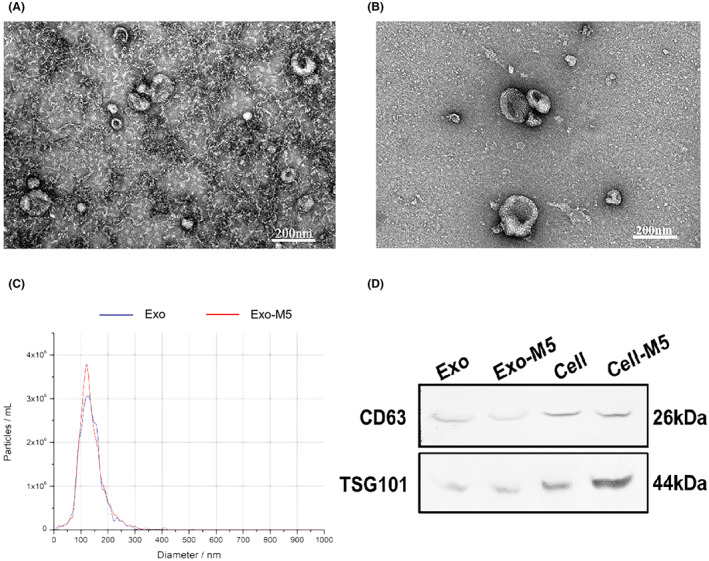
Purification and identification of exosomes secreted from *Brucella melitensis* M5‐infected macrophages. (A and B) Method of the isolation and purification of exosomes from cell culture supernatant based on differential centrifugation. The purified exosomes secreted from *Brucella*‐infected macrophages (EXO‐M5) and secreted from macrophages (EXO) were analysed by transmission electron microscopy (TEM). (C) The EXO‐M5 and EXO size distributions were tested by nanoparticle tracking analysis (NTA). (D) Western blotting was used to identify exosomal markers (CD63, TSG101) in exosomes (5 μg/well) derived from uninfected or *B. melitensis* M5‐infected macrophages and in the corresponding whole cell lysates.

Using nanoparticle tracking analysis (NTA) to detect nanoparticle sizes and transmission electron microscope (TEM) observation, we found that the exosomes collected from *B. melitensis* M5‐infected macrophages (EXO‐M5; Figure 1A) or the exosomes collected from macrophages (EXO; Figure 1B) were saucer‐like three‐dimensional double‐layer membrane structures with diameters of 30–120 nm (Figure 1C). The number of the EXO‐M5 was slightly more than that of the EXO. The background was clean and conformed to the definition of exosomes.

Western blotting detected the surface markers CD63 and TSG101 on exosomes, and exosomes released from macrophages infected with *B. melitensis* M5 positively expressed specific markers (Figure 1D).

### Exosomes promote M1 polarization in macrophages by carrying *Brucella*

**FIGURE 2:**
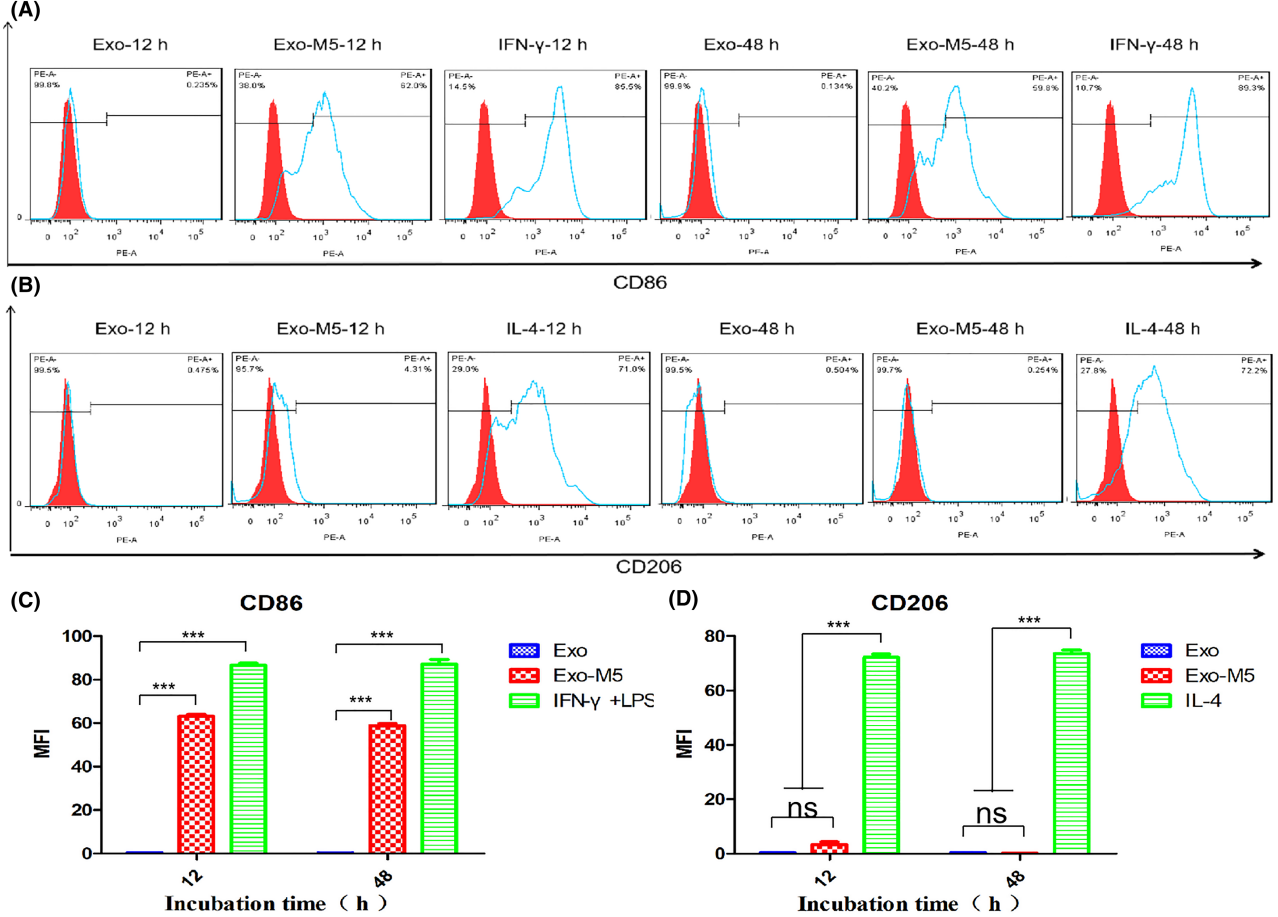
Flow cytometric analysis of the expression of M1/M2 surface marker molecules (CD86/CD206) in Exo and Exo‐M5 incubated macrophages. The cells incubated with Exo, Exo‐M5, IFN‐γ and LPS or IL‐4 (control group) for 12 and 48 h and polarized macrophages were counted via flow cytometry. CD86 (A and C) and CD206 (B and D) were used as makers to identify M1‐type or M2‐type macrophages. All treatments were repeated three times with *n* = 3/time point. Statistical significance was determined by one‐way ANOVA. Data were presented as means ± SD. ***p < 0.001, ^ns^ *p* > 0.05.

In order to test whether exosomes bearing *B. melitensis* are associated with signalling and promoting polarization in macrophages, macrophages derived from bone marrow were incubated with Exo or Exo‐M5 for 12 and 48 h and then the expression levels of macrophage marker molecule M1 CD86 and M2 CD206 were measured using flow cytometry analysis. Furthermore, macrophages incubated with IFN‐γ and LPS or IL‐4 were used as positive controls to stimulate macrophage M1/M2 polarization. We found that the Exo‐M5 group significantly induced CD86 expression (Figure 2A,C) and promoted macrophage polarization of the M1 type, but Exo did not (Figure 2B,D).

### Exosomes promote the expression of M1‐type macrophage‐related factors by carrying *Brucella*

**FIGURE 3:**
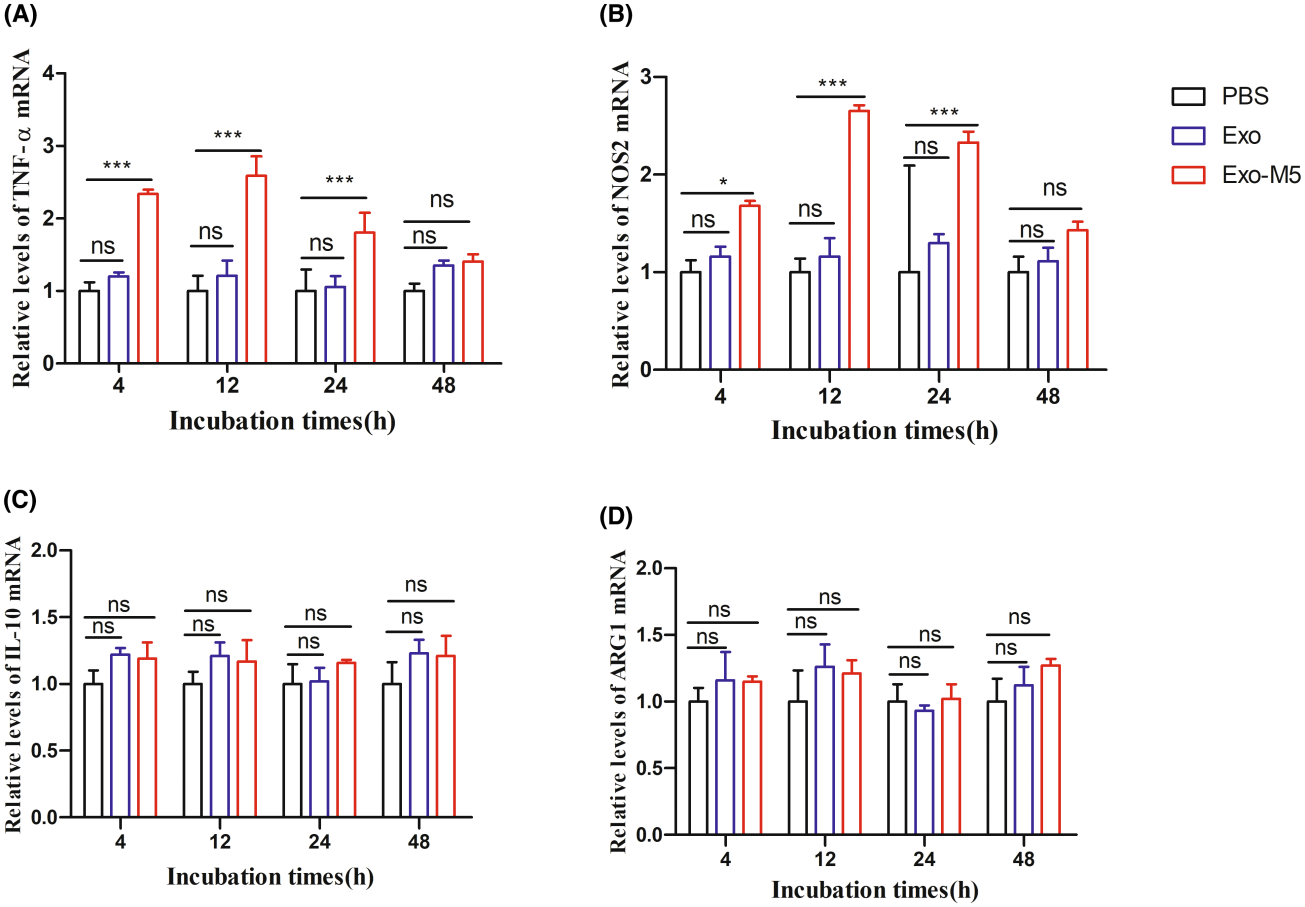
Exosomes derived from *Brucella melitensis* M5‐infected macrophages promote M1‐related factors mRNA transcription in macrophages. The transcription level of TNF‐α (A), NOS2 (B), IL‐10 (C) and ARG1 (D) were assessed using the qRT‐PCR. PBS‐treated samples were used as a control group. All treatments were repeated three times with *n* = 3/time point. Data were presented as means ± SD. Statistical significance was determined by one‐way ANOVA. *p < 0.05. ***p < 0.001. ^ns^ *p* > 0.05.

To explore the effects of exosomes on the transcriptional expression of M1/M2‐related factors in macrophages, we used qRT‐PCR to detect the M1‐related factors TNF‐α and NOS2 and the M2‐related factors IL‐10 and ARG1 induced by exosomes. Compared with the phosphate‐buffered saline (PBS), Exo‐M5 group significantly induced the transcription of TNF‐α and NOS2 at 4, 12 and 24 h time points examined (*p* < 0.05 or *p* < 0.001, Figure 3A,B), which reached a maximum at 12 h. In addition, Exo‐M5 did not significantly induce the expression of IL‐10 and ARG1 (*p* > 0.05, Figure 3C,D). Exo group has no effect on the release of M1‐ and M2‐related factors. This confirms that exosomes can promote the expression of M1‐type macrophage marker‐related factors by carrying *Brucella*.

### Exo‐M5 regulates the activation of M1/M2 molecular signalling pathway

**FIGURE 4:**
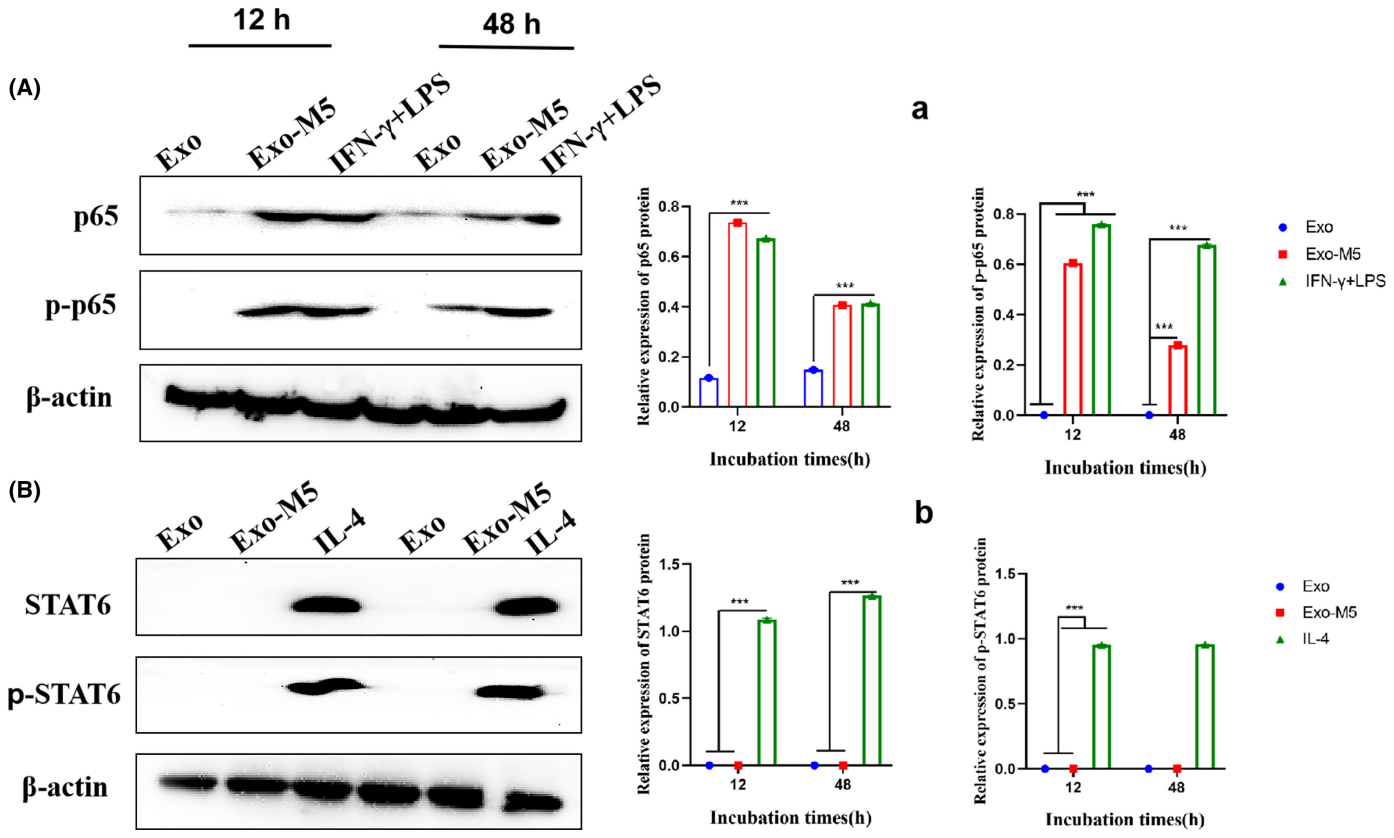
Exosomes released from *Brucella melitensis* M5‐infected cells (Exo‐M5) induce the activation of NF‐κB p65 signalling pathway. Exosome were incubated with macrophage for 12 h and 48 h. Western blot analysis of p65, p‐p65 (A, a) and STAT6, p‐STAT6 protein expression in exosome (B, b). All treatments were repeated three times with *n* = 3/time point. Data were presented as means ± SD. Statistical significance was determined by one‐way ANOVA. ***p < 0.001.

To detect whether Exo‐M5 regulates macrophage polarization through the macrophage polarization‐related signalling pathway, western blotting was used to detect activation of the M1/M2 gene signalling pathways. Exo‐M5 activated the M1 signalling pathway via NF‐κB p65 and phosphorylated p‐p65, which was time dependent and reached a peak at 12 h (*p* < 0.001, Figure 4A). However, Exo‐M5 did not activate the M2 type signal pathway via STAT6 and p‐STAT6 (*p* > 0.05, Figure 4B). These results show that infectious exosomes can induce the activation of NF‐κB pathway, which may be closely related with M1‐type macrophages.

### Detection of *Brucella* intracellular survival

**FIGURE 5:**
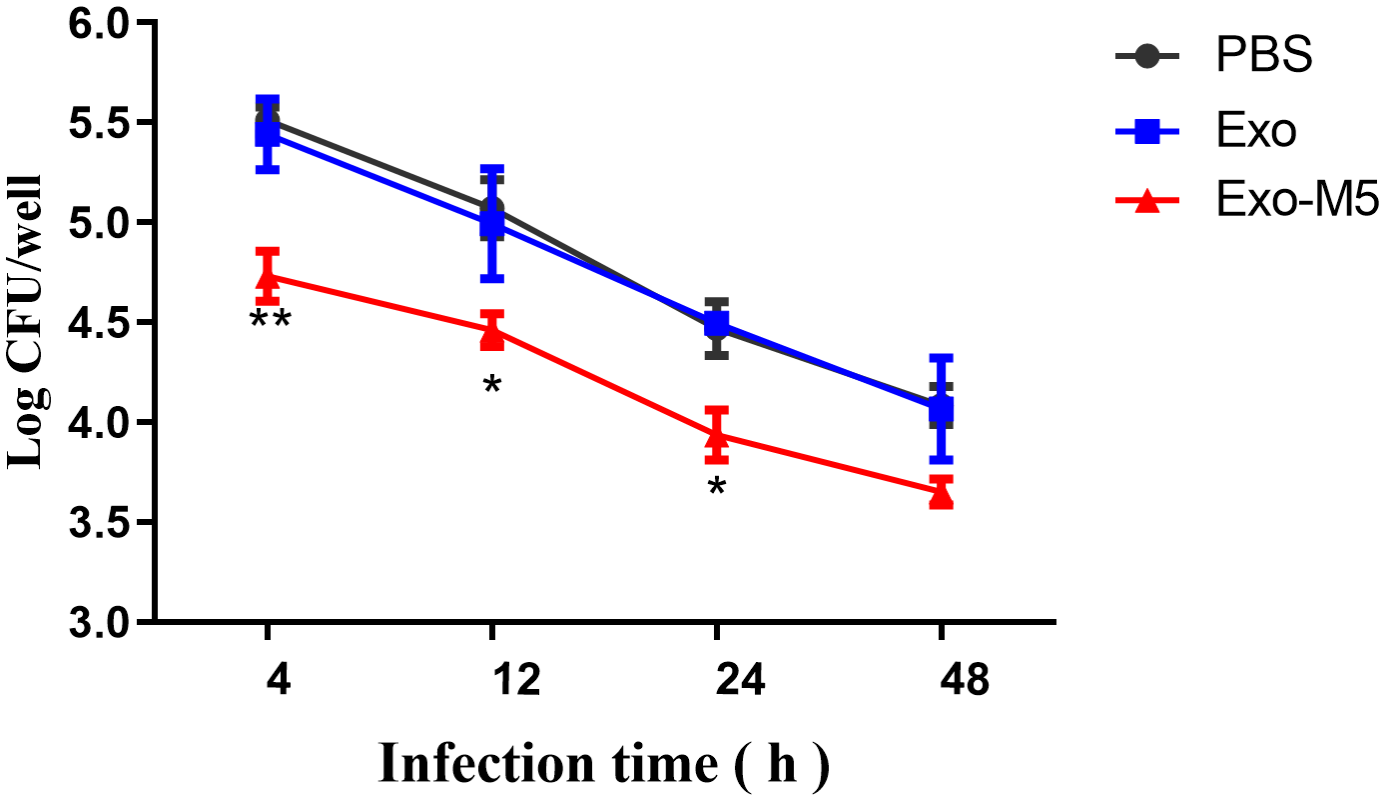
The intracellular bacterial survival was tested by measuring CFU. Recipient cells pre‐treated with Exo‐M5 or Exo, PBS as a control group and then the CFU level in macrophages were measured at different time points post‐infection. All treatments were repeated three times with *n* = 3/time point. Data were presented as means ± SD. Statistical significance was determined by one‐way ANOVA. *p < 0.05. **p < 0.01.

To explore the effect of exosome‐induced macrophage polarization on the intracellular survival of *Brucella*, we used *B. melitensis* M5 to infect macrophages pre‐treated with exosomes and counted intracellular colony‐forming units (CFUs) at different time points. The results showed that at 4, 12 and 24 h of *B. melitensis* M5 infection, Exo‐M5 pre‐treated macrophages significantly inhibited the intracellular CFUs of *B. melitensis* M5 (*p* < 0.05 or *p* < 0.01, Figure 5) compared with the PBS control group and that this was time dependent with a gradual downward trend overall. The results confirmed that exosome‐induced M1‐type macrophages inhibited the intracellular survival of *Brucella* and that the secretion of Th1 type cytokines may be a key factor in eliminating intracellular bacteria.

### Detection of the relevant immune index in mice

**FIGURE 6:**
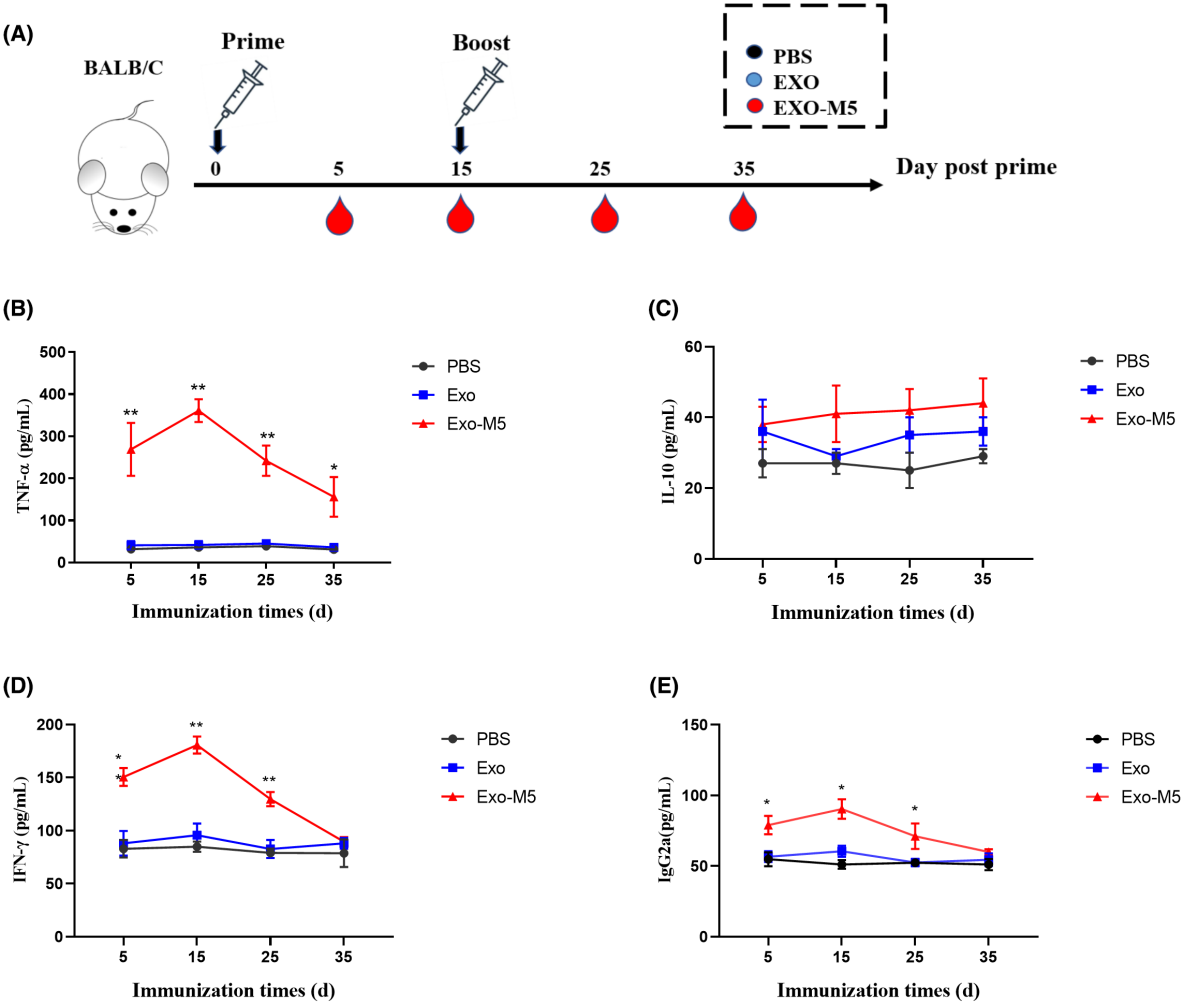
Immune effects of exosomes in mice. Schematic diagram of the immunization schedule and overall experimental design (A). The mouse serum were harvested and TNF‐ɑ (B), IL‐10 (C), IFN‐γ (D) and IgG2a (E) production (pg/ml) were assessed by ELISA assay. All treatments were repeated three times with *n* = 3/time point. Data were presented as means ± SD. Statistical significance was determined by one‐way ANOVA. *p < 0.05. **p < 0.01.

To determine whether *Brucella*‐infected exosomes affected the release of M1 (TNF‐α) and M2 (IL‐10) cytokines in mice, we immunized mice with exosomes for 5, 15, 25 and 35 days and then measured TNF‐α, IL‐10 and IgG2a protein levels (Figure 6A). Results showed that *Brucella*‐infected exosomes significantly induced the release of TNF‐α compared with the PBS control group, and reached a maximum at 12 days after immunization (*p* < 0.05 or *p* < 0.01, Figure 6B), but had no effect on the secretion of IL‐10 (*p* > 0.05, Figure 6C). However, exosomes released by uninfected macrophages did not induce macrophage polarization in vivo.

The Exo‐M5 group significantly induced the release of IFN‐γ and IgG2a (*p* < 0.05 or *p* < 0.01, Figure 6D,E) on days 5, 15 and 25 after immunization compared with the PBS control group. However, 35 days after immunization, the ability of Exo‐M5 to induce innate immunity gradually decreased. This suggests that Exo‐M5 induces high levels of Th1‐type immune responses in mice.

### Effect of exosomes on immune protection in mice

**FIGURE 7:**
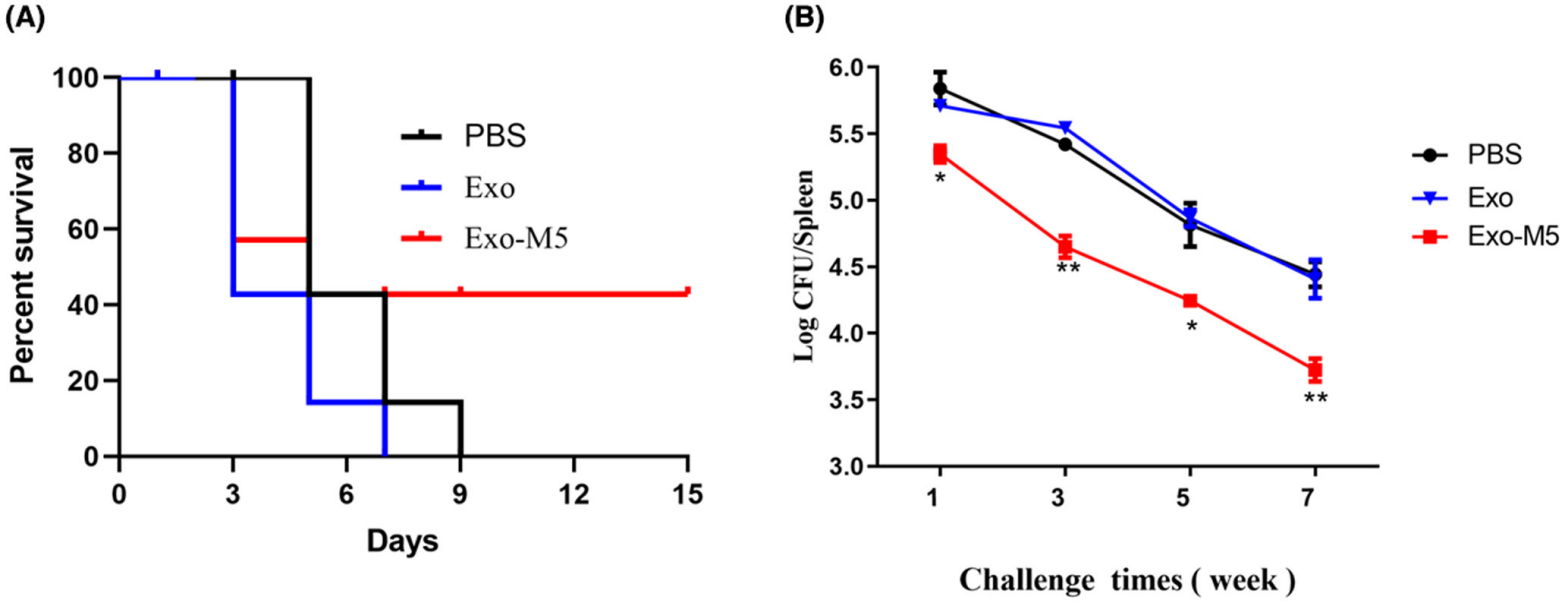
Mice immunized with Exo‐M5 were protected against *Brucella* infection. Mice immunized with different groups of exosomes were infected with *B. melitensis* M5 (1 × 10^9^) for 15 days to observe the survival rate of mice in different groups. Mice immunized with different groups of exosomes were infected with *B. melitensis* M5 (1 × 10^6^) for 1, 3, 5 and 7 weeks infection. Then, each group of mice was sacrificed and mice spleens CFU (B) were measured. All treatments were repeated three times with *n* = 3/time point. Data were presented as means ± SD. Statistical significance was determined by one‐way ANOVA. *p < 0.05. **p < 0.01.

Next, we investigated whether *Brucella*‐infected exosome‐mediated macrophage polarization affected the survival rate and spleen CFUs of mice after an injection of *B. melitensis* M5. Mice in different immunization groups were infected with a maximum dose of 1 × 10^9^ CFUs 35 days after immunization, and the survival of the mice was recorded. Nine days after *Brucella* infection, the survival rate of mice in the PBS and Exo groups was 0%, whereas that of mice in the Exo‐M5 group was 42.8% (Figure 7A). Therefore, macrophage exosomes infected by *B. melitensis* M5 improved the resistance of mice against *Brucella* infection, thereby improving their survival rate.

At 35 days after Exo and Exo‐M5 immunization, the spleens of mice in each group were isolated at 1, 3, 5 and 7 weeks after *B. melitensis* M5 (1 × 10^6^) infection and the spleen CFU count of each group was calculated. The results showed that compared with the PBS group, the amount of bacteria in the spleens of mice in the Exo‐M5 group was significantly reduced at each time point (*p* < 0.05 or *p* < 0.01, Figure 7B). There was no significant difference in the Exo groups at each time point. In conclusion, macrophage exosomes infected by *B. melitensis* M5 reduced damage to the spleen, to varying degrees, caused by *Brucella* infection. Exosomes also reduced the survival of *Brucella* in mice, and the combined adjuvant was beneficial for improving the immune protective effect of the exosomes.

### Identification of *Brucella* components in exosomes

**FIGURE 8:**
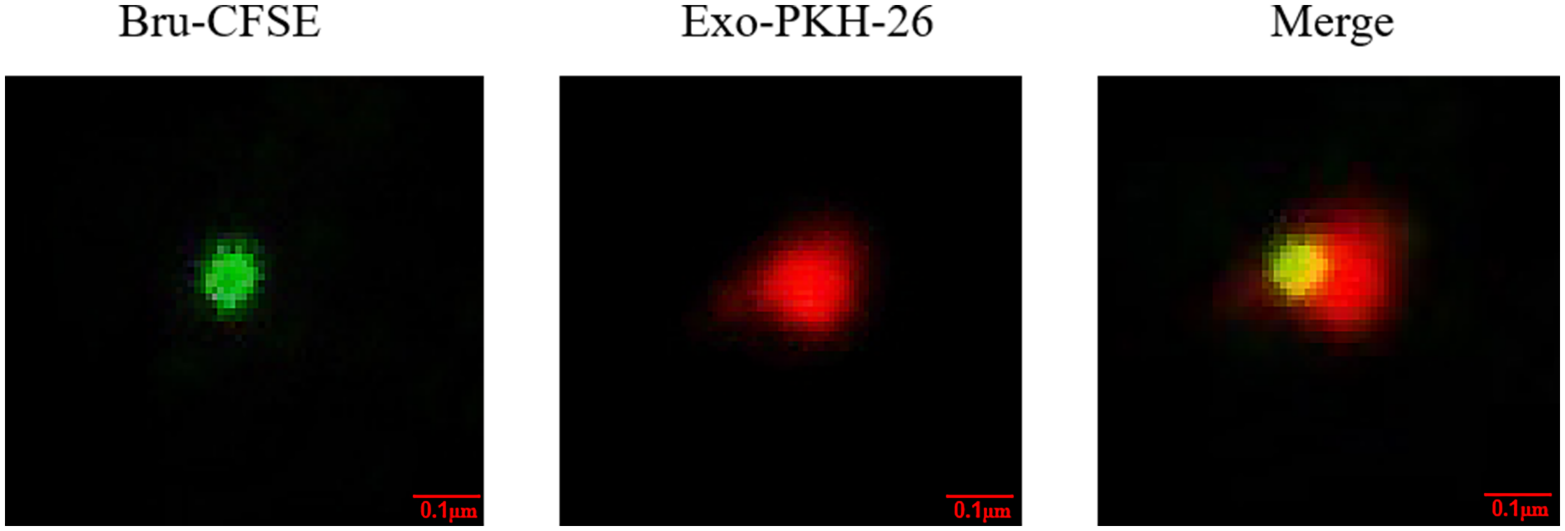
Detection of *Brucella melitensis* M5 in microvesicles by confocal microscopy. Macrophages were infected with *Brucella* labelled with CFSE, then the exosomes in the culture medium were extracted and labelled with PKH‐26. Bru‐CFSE: CFSE‐labelled *Brucella* components (green fluorescence); Exo‐PKH‐26: PKH‐26‐labelled exosomes released from *Brucella*‐infected macrophages (red fluorescence); Exo‐M5: exosomes released from *Brucella*‐infected macrophages RAW264.7.

After 56‐carboxyfluorescein diacetatesuccinimidyl ester (CFSE)‐labelled *B. melitensis* M5 and PKH26‐labelled macrophages were co‐cultured, exosomes were extracted and observed with a confocal microscope. Red‐stained microparticles contained green‐stained *B. melitensis* M5 components (Figure 8), indicating that RAW264.7 macrophages lysed *Brucella* after phagocytosis and that some of the lysed bacterial components were released extracellularly through the exosome pathway.

### Analysis of protein aggregation in exosomes

Next, we investigated the components in the RAW264.7 macrophage exosomes. Label‐free quantitation technologies were used to analyse the protein profiles of exosomes secreted from macrophages (with or without *Brucella* infection). Overall, 1236 proteins were identified, including 1156 proteins derived from host cells and 80 proteins derived from *B. melitensis* M5. Particularly, we found that 80 proteins contained in infectious exosomes were specific to *B. melitensis* M5, including outer membrane proteins, LPS‐related proteins, transporters and other proteins (Table 1). Thus, *Brucella* antigens in these exosomes may be important stimulators that mediate macrophage polarization.

**TABLE 1:** List of major *Brucella melitensis* M5 antigen proteins in exosomes.

Protein ID	Protein name	Gene name	Peptides
P0A3U4	31 kDa outer membrane immunogenic protein	Omp31	2
A0A0F6AQ26	OmpA‐like transmembrane domain protein	BAbS19_I06750	4
A0A0E1WXV9	ABC transporter domain‐containing protein	BALG_02952	2
D6LMH8	TRAP dicarboxylate transporter	BAZG_00645	2
C0G8E8	ABC transporter, ATP‐binding protein	BCETI_6000119	3
A0A0F6AP74	ATP/GTP‐binding site motif A (P‐loop)	BAbS19_I03340	2
A0A0E1X306	Import inner membrane translocase subunit Tim44	BALG_01503	2
C0G3P7	Porin subfamily protein	BCETI_1000291	2
A7WNU7	Outer membrane protein 25b	Omp25b	2
A0A0U5MYX3	Transcriptional regulator NnrR	BF3285c2_0933	2
A0A4Q5MJR3	Crp/Fnr family transcriptional regulator	EVD24_16430	2
D6LM58	Chaperone protein DnaK	dnaK	2
E2PKN0	19 kDa periplasmic protein	BIBO2_0596	3

## DISCUSSION

Macrophages, an important component of immune responses, remove pathogens and maintain an immune steady state. Macrophages have strong plasticity and after a host is stimulated by pathogenic microorganisms, they can differentiate into different subtypes (M1/M2), which have different biological functions (Wang et al., 2010). M1‐type macrophages mediate Th1‐type immune response and promote the expression of M1‐type‐related factor NO and Th1 cytokines such as TNF‐α, IL‐6 and IL‐12, whereas M2 macrophages mediate Th2‐type immune responses and release Th2 cytokines such as IL‐4 and IL‐10. M1‐ and M2‐type macrophages can also be transformed in a special microenvironment (Verreck et al., 2004). However, macrophages are important *Brucella* host cells and *Brucella* was reported to polarize macrophages to an M1 type in the early stages of infection, leading to increased levels of the Th1 immune factor IFN‐γ and a rapid decrease in the bacterial load of the mouse spleen (Konrad et al., 2005).

Exosomes are an important medium for transmitting material information, and they have functions in tumours, autoimmune diseases and pathogenic microorganism infections. Although research on the role of exosomes in pathogenic microbial infections is in the preliminary stage, exosomes might have great potential for the prevention and treatment of infectious diseases. Previous studies found that *M. tuberculosis* induced immune cells to produce a large number of exosomes. In this study, we discovered that *B. melitensis*, an intracellular bacteria, induced macrophages to release exosomes by a time‐dependent mechanism. Exosomes are important substance signal carriers released by cells, and increasing numbers of studies have reported that exosomes affected the biological functions of macrophages by regulating the polarization of macrophages. A study by Moradi‐Chaleshtori et al. (2021) showed that exosome‐mediated miR‐33 transfer induced M1 polarization in mouse macrophages and exerted antitumor effects in a 4T1 breast cancer cell line. Cheng et al. (2017) found exosomes from M1‐polarized macrophages potentiate the cancer vaccine by creating a pro‐inflammatory microenvironment in the lymph node.

In this study, we found that exosomes secreted from *B. melitensis* M5‐infected macrophages induced bone marrow‐derived mononuclear macrophages to polarize to an M1 type and promoting the release of Th1‐type immune factors (NOS2, TNF‐α). NF‐κB is an important immunogenic regulatory pathway that regulates the secretion of the pro‐inflammatory cytokines IL‐1β, IL‐6 and IL‐12 among others (Shah et al., 2011). Some studies have found that many pathogenic microbial infections are able to promote M1 polarization of macrophages by activating the Akt, mTOR and NF‐κB pathway (Gary & Elizabeth, 2009; Lin et al., 2018); the activation of NF‐κB pathway by Exo‐M5 was also detected in our experiments, suggesting that Exo‐M5 may mediate M1 polarization of macrophages/monocytes via NF‐κB activation. In addition, LPS and IFN‐γ are important activators of macrophages and their combination can strongly induce the activation of NF‐κB pathway; therefore, many scholars believe that the activation of NF‐κB signalling pathway can promote the secretion of Th1‐type cytokines (Chow et al., 2012). Studies suggested that Th1‐type factors can induce immune cells to produce reactive oxygen species (ROS), which can directly kill pathogenic microorganisms and help mobilize natural killer cells to perform important antibacterial functions (Jiang et al., 1993). An increase in the ROS concentration is disadvantageous to the intracellular proliferation of *Brucella* (Li et al., 2016). Our study found that exosome‐induced M1 macrophages inhibited the intracellular survival of *Brucella*, indicating Th1 cytokine‐mediated ROS release may be an important factor in the inhibition of *Brucella* reproduction. In addition, animal experiments demonstrated that exosomes induced the release of M1 cytokines in vivo, thereby reducing the number of *Brucella* in the spleen, by a mechanism that might involve M1‐type cytokines (IFN‐γ, IL‐12) released by M1 macrophages that directly kill *Brucella* in the host, as well as enhance the phagocytosis and bactericidal ability of macrophages, which can rapidly clear *Brucella* from the host (Xu et al., 2021). Therefore, exosome‐mediated macrophage polarization is a new means of removing *Brucella* from a host. Exosomes are important carriers of proteins, nucleic acids and lipids, and allow the transfer of information between cells (Mathivanan et al., 2010).

Exosomes carrying bacterial antigens have an important role in the host immune response. In this study, we confirmed that the exosomes were loaded with a critical antigenic protein from *Brucella* that affected the polarization of macrophages. We showed that *Brucella* antigens, indicated by green fluorescence, were present in exosomes released from macrophages using confocal laser scanning microscopy. Furthermore, we also found 80 important *Brucella* antigens using label‐free protein histology, including Omp31, Omp19, Omp25b, L7/L12, Cu/Zn‐SOD and others in exosomes released by *Brucella‐*infected macrophages. Therefore, these proteins in the exosomes may be an important factor in inducing macrophage polarization. Omp31 and Omp25B are immunogenic and were reported to induce humoral and cellular immune responses, as well as have protective effects against *Brucella* (Clausse et al., 2013). A study reported that mice immunized with peptides of OMP31 containing T epitope, B epitope or TB epitopes are of high immunogenicity, which can protect mice from *B. melitensis* infection in the lung (Zhang et al., 2019) and that a stable memory immune response was produced (Ranjbar et al., 2016). Therefore, exosomes containing antigens can inhibit the reproduction of *Brucella* in mice and induce the M1 polarization of macrophages in vivo to promote Th1‐type immunity. The secretion of cytokines, such as TNF‐α and IFN‐γ, inhibit the replication of bacteria and exosomes carrying antigens can induce the host to produce IgG, resulting in protection against infection. However, how the antigen molecules in the exosomes interact with the host and how they activate the immune response mechanisms require further exploration.

In summary, our study confirmed that exosomes released by *B. melitensis* M5‐infected macrophages induced bone marrow‐derived mononuclear macrophages to polarize to M1 and promoted the secretion of Th1 cytokines, thereby indirectly inhibiting the survival of *B. melitensis* M5 in vivo and in vitro. In addition, exosomes stimulated strong immune responses in mice that were protective against infection. These immunostimulatory effects may be related to *Brucella* antigens present in the exosomes. Taken together, these findings provide a basis to explore the role of exosomes in host immune responses and aid the development of new *Brucella* vaccines.

### Bacterial strains, cells and mice

*Brucella melitensis* M5 was provided by the Chinese Center for Disease Control and Prevention. RAW264.7 macrophages were obtained from the Type Culture Collection of the Chinese Academy of Sciences (China, Beijing). BALB/c mice aged 6 weeks were purchased from the Experimental Animal Center of Xinjiang Medical University. All animal care in this study was carried out in accordance with institutional animal care guidelines and related laws.

### Preparation of mouse bone marrow‐derived mononuclear macrophages

Bone marrow cells were isolated from mouse femurs and tibias and cultured in RPMI 1640 medium. On the second day, mouse M‐CSF (RPMI 1640 + 10% FBS + M‐CSF) was added at a concentration of 10 ng/ml to induce bone marrow monocytes to differentiate into monocytes/macrophages. On the seventh day, the differentiated bone marrow‐derived mononuclear macrophages were collected.

### *Brucella melitensis* M5 infection of RAW264.7 macrophages

RAW264.7 macrophages were infected with *B. melitensis* M5 for 1 h at a multiplicity of infection (MOI; bacteria: cell) of 100:1. Cells were incubated for 1 h and then washed three times with PBS. Next, gentamicin (25 μg/ml) was added into the cell culture medium and incubated for 45 min to kill *Brucella* outside the cells. The culture medium was discarded and replaced. At 24 h post‐*Brucella* infection, the supernatants were collected from each plate.

### Extraction and identification of exosomes

The extraction and identification methods for exosomes were reported previously (Chen et al., 2020). RAW264.7 macrophages were infected with *B. melitensis* M5 and cultured at an MOI of 100:1 (bacteria/cell) for 1 h. After adding gentamicin sulphate for 30 min, the cell culture supernatant was collected at 24 h after infection. Then, supernatants were centrifuged at 1000 × *g* for 5 min, 5000 × *g* for 5 min and 14,000 × *g* for 30 min to eliminate the cell debris. The supernatant was passed through a 0.22‐μm filter to eliminate *Brucella* and then centrifuged further for 2 h at 12,000 × *g* to pellet the exosomes. *Brucella* uninfected cells were also used as controls to extract exosomes using the same procedure.

The purified exosomes were identified by NTA, TEM and western blotting as described by Chen (Chen et al., 2020). The total protein concentration of exosomes was measured using a BCA assay kit (Pierce, Rochford, IL). Before the experiment, all isolated exosomes were stored at −80°C.

### Flow cytometric analysis

The bone marrow‐derived mononuclear macrophages were incubated with exosomes, and then macrophages were collected at 12 and 48 h. In addition, IFN‐γ and LPS or IL‐4‐stimulated cells were added to the cells as a positive control. The expressions of the M1‐type macrophage marker molecule CD86 and M2‐type macrophage marker molecule CD206 were detected by flow cytometry with IgG2a as an isotype control. After the macrophages of different treatment groups were fixed and ruptured, the corresponding antibodies were added, incubated, protected from light at 4°C for 30 min, washed with 1× binding buffer and tested on the flow cytometer.

### Real‐time PCR

Exosomes were co‐incubated with 5 mmol L^−1^ ATP and peritoneal macrophages or bone marrow‐derived mononuclear macrophages. At 8 and 24 h, Trizol lysate was added, RNA was extracted according to the RNA extraction kit instructions and the total RNA concentration was measured. Then, a cDNA synthesis kit was used for the reverse transcription of the total RNA.

A fluorescence real‐time quantitative PCR instrument was used to detect the expression levels of mRNAs for M1/M2‐related genes. The RT‐PCR system included 1 μl cDNA, 5 μl SYBR green mix, 0.2 μl of upstream and downstream primers and 3.6 μl sterile water. The reaction conditions were pre‐denaturation at 95°C for 30 s, denaturation at 95°C for 30 s, extension at 60°C for 35 s, with 45 cycles. *GAPDH* was used as an internal control.

### Western blotting

Bone marrow‐derived mononuclear macrophages and peritoneal macrophages were incubated with exosomes, and protein samples were collected at 12 and 48 h. In addition, IFN‐γ and LPS or IL‐4 stimulated cells were added to the cells as a positive control. Western blotting was used to detect the protein expressions of NF‐κB p65, p‐p65 and STAT6, p‐STAT6. All collected protein samples were tested for related protein expression levels according to the above‐mentioned western blotting method.

### CFU detection

Exosomes released from different pre‐treated cells were incubated with bone marrow‐derived mononuclear macrophages for 8 h and then infected with *B. melitensis* M5 for 4, 8, 12, 24 or 48 h. Then, these cells were lysed with 0.5 ml of 0.2% Tween 20 at each of post‐infected time point, incubated for 15–30 min on ice, followed by rinsing each well with 0.5 ml of PBS. Viable bacteria were quantified by serial dilution in sterile PBS and plating on TSA and then they were incubated at 37°C for 72 h followed by colony counting.

### Immunization of mice with exosomes

Three immunization groups were formed: exosomes released from *Brucella*‐infected RAW264.7 macrophages combined with adjuvant (Exo‐M5); exosomes released from normal RAW264.7 macrophages combined with adjuvant (Exo) and PBS alone. The immunization dose for each group was 100 μl (0.3 μg/μl). Immunization was performed two times at 15 days intervals and 100 μl was injected each time.

### *Brucella* challenge

Mice were inoculated with exosomes 35 days later. The maximum lethal dose of *B. melitensis* M5 (1 × 10^9^ CFUs) was suspended in 0.1 ml PBS and then injected subcutaneously into the abdominal cavity of mice. The survival of mice and the mental state of the surviving mice were noted. Of note, 0.1 ml of PBS was injected as a control. Mice were inoculated with exosomes 35 days later and the optimal dose of *B. melitensis* M5 was injected into the abdominal cavity of the mice.

### Detection of the immune index and measurement of the spleen CFUs in mice

On days 5, 15, 25 and 35 after immunization, the mice in each group were tail‐clamped to collect blood and serum. Mouse IgG2a and IFN‐γ ELISAs were performed in accordance with the manufacturer's instructions. Three mice in each group were sacrificed by cervical dislocation, spleens were removed and spleen cells were isolated and counted. The number of spleen lymphocytes was adjusted to 1 × 10^6^ cells/ml and they were added to a 96‐well plate. Then, 10 μl of inactivated *B. melitensis* M5 (10^7^ CFUs/well) was added for 68 h. Next, the lymphocyte culture supernatant was collected and used for mouse IFN‐γ ELISA.

At 5, 15, 25 and 35 days after *B. melitensis* M5 infection, three *Brucella*‐infected mice per group were humanely sacrificed, and spleens were removed and weighed. The spleen was placed into a centrifuge tube containing PBS buffer, 1 ml of 0.2% Triton X‐100, several small steel balls and homogenized in a homogenizer. Then, 100 μl of the diluted homogenate was diluted in sterile saline and plated onto TSA. The plates were incubated at 37°C and *Brucella* CFUs were enumerated. The results were presented as log_10_ CFUs per spleen.

### Chemical staining

CFSE (Sigma, USA)‐stained *B. melitensis* M5 was added to PKH26 (Sigma, USA)‐stained RAW264.7 macrophages and mixed. *Brucella* particles were added at 50 times the number of cells. After 12 h of co‐cultivation, the supernatant was collected by centrifugation and the staining results were observed under a confocal microscope.

### Exosomal proteomics analysis

In this experiment, the identified exosomes were sent to Applied Protein Technology Co., Ltd. (Shanghai, China) for label‐free quantitative proteomics analysis. The exosome samples were divided into two groups: exosomes released from normal RAW264.7 macrophages (uninfected group) and exosomes released from *Brucella* infected RAW264.7 macrophages (infected group), with three replicates per group.

### Data analyses

All original data were analysed using Microsoft Excel software, and GraphPad Prism statistical software was used for graphing and one‐way analysis of variance. The results are displayed using the mean ± standard deviation (M ± SD), and differences were considered statistically significant when *p* values were <0.05 and <0.01.

## AUTHOR CONTRIBUTIONS

**Wang Yueli:** Conceptualization (equal); formal analysis (equal); methodology (equal); supervision (equal); validation (equal); writing – original draft (equal); writing – review and editing (equal). **Honghuan Li:** Data curation (equal); methodology (equal); resources (equal); validation (equal). **Zhenyu Xu:** Validation (equal); writing – review and editing (equal). **Jihai Yi:** Data curation (equal); methodology (equal); validation (equal); writing – review and editing (equal). **Wei Li:** Methodology (equal); resources (equal); validation (equal). **Chuang Meng:** Validation (equal); writing – review and editing (equal). **Huan Zhang:** Formal analysis (equal); validation (equal). **Xiaoyu Deng:** Data curation (equal); formal analysis (equal); resources (equal); validation (equal). **Zhongchen Ma:** Validation (equal); visualization (equal); writing – original draft (equal); writing – review and editing (equal). **Yong Wang:** Validation (equal); writing – review and editing (equal). **Chuangfu Chen:** Validation (equal); writing – review and editing (equal).

## CONFLICT OF INTEREST STATEMENT

The authors have declared that there are no competing interests.

## References

[mbt214274-bib-0001] Chen, P. , Ruan, A. , Zhou, J. , Huang, L. , Zhang, X. , Ma, Y. et al. (2020) Extraction and identification of synovial tissue‐derived exosomes by different separation techniques. Journal of Orthopaedic Surgery and Research, 15, 1–8. 32151262 10.1186/s13018-020-01604-x PMC7063768

[mbt214274-bib-0002] Cheng, L. , Wang, Y. & Huang, L. (2017) Exosomes from M1‐polarized macrophages potentiate the cancer vaccine by creating a pro‐inflammatory microenvironment in the lymph node. Molecular Therapy, 25, 1665–1675. 28284981 10.1016/j.ymthe.2017.02.007 PMC5498801

[mbt214274-bib-0003] Chow, Y. , Lee, K. , Vidyadaran, S. , Lajis, N.H. , Akhtar, M.N. , Israf, D.A. et al. (2012) Cardamonin from *Alpinia rafflesiana* inhibits inflammatory responses in IFN‐γ/LPS‐stimulated bv2 microglia via NF‐κB signalling pathway. International Immunopharmacology, 12, 8–9. 10.1016/j.intimp.2012.01.009 22306767

[mbt214274-bib-0004] Clausse, M. , Díaz, A.G. , Ghersi, G. , Zylberman, V. , Cassataro, J. , Giambartolomei, G.H. et al. (2013) The vaccine candidate BLSOmp31 protects mice against *Brucella canis* infection. Vaccine, 31, 6129–6135. 23906889 10.1016/j.vaccine.2013.07.041

[mbt214274-bib-0005] Dai, Y. , Wang, S. , Chang, S. , Ren, D. , Shali, S. & Li, C. (2020) M2 macrophage‐derived exosomes carry microRNA‐148a to alleviate myocardial ischemia/reperfusion injury via inhibiting TXNIP and the TLR4/NF‐κB/NLRP3 inflammasome signaling pathway. Journal of Molecular and Cellular Cardiology, 142, 65–79. 32087217 10.1016/j.yjmcc.2020.02.007

[mbt214274-bib-0006] Gao, W. , Liu, H. , Yuan, J. , Wu, C. , Huang, D. , Ma, Y. et al. (2016) Exosomes derived from mature dendritic cells increase endothelial inflammation and atherosclerosis via membrane TNF‐α mediated NF‐κB pathway. Journal of Cellular and Molecular Medicine, 20, 2318–2327. 27515767 10.1111/jcmm.12923 PMC5134386

[mbt214274-bib-0007] Garcia‐del Portillo, F. , Stein, M.A. & Finlay, B.B. (1997) Release of lipopolysaccharide from intracellular compartments containing *Salmonella typhimurium* to vesicles of the host epithelial cell. Infection and Immunity, 65, 24–34. 8975888 10.1128/iai.65.1.24-34.1997 PMC174552

[mbt214274-bib-0008] Gary, C. & Elizabeth, R. (2009) NF‐κB and phosphatidylinositol 3‐kinase activity mediates the HCMV‐induced atypical M1/M2 polarization of monocytes. Virus Research, 4, 26. 10.1016/j.virusres.2009.04.026 PMC2736317 19427341

[mbt214274-bib-0009] Giri, P.K. & Schorey, J.S. (2008) Exosomes derived from *M. bovis* BCG infected macrophages activate antigen‐specific CD4+ and CD8+ T cells in vitro and in vivo. PLoS One, 3, e2461. 18560543 10.1371/journal.pone.0002461 PMC2413420

[mbt214274-bib-0010] Higuchi, H. , Shoji, T. , Murase, Y. , Iijima, S. & Nishijima, K.I. (2016) Siglec‐9 modulated IL‐4 responses in the macrophage cell line RAW264. Bioscience, Biotechnology, and Biochemistry, 80, 501–509. 26540411 10.1080/09168451.2015.1104238

[mbt214274-bib-0011] Jiang, X. , Leonard, B. , Benson, R. & Baldwin, C.L. (1993) Macrophage control of *Brucella abortus*: role of reactive oxygen intermediates and nitric oxide. Cellular Immunology, 151, 309–319. 8402938 10.1006/cimm.1993.1241

[mbt214274-bib-0012] Konrad, D. , Rudich, A. , Bilan, P.J. , Patel, N. & Richardson, C. (2005) Troglitazone causes acute mitochondrial membrane depolarisation and an AMPK‐mediated increase in glucose phosphorylation in muscle cells. Diabetologia, 48, 954–966. 15834551 10.1007/s00125-005-1713-7

[mbt214274-bib-0013] Li, T. , Xu, Y. , Liu, L. , Huang, M. , Wang, Z. & Tong, Z. (2016) *Brucella melitensis* 16M regulates the effect of AIR domain on inflammatory factors, autophagy, and apoptosis in mouse macrophage through the ROS signaling pathway. PLoS One, 11, 1–22. 10.1371/journal.pone.0167486 PMC5132199 27907115

[mbt214274-bib-0014] Lin, L.R. , Gao, Z.X. , Lin, Y. , Zhu, X.Z. , Liu, W. , Liu, D. et al. (2018) Akt, mTOR and NF‐κB pathway activation in *Treponema pallidum* stimulates m1 macrophages. International Immunopharmacology, 59, 181–186. 29656208 10.1016/j.intimp.2018.03.040

[mbt214274-bib-0015] Mathivanan, S. , Ji, H. & Simpson, R.J. (2010) Exosomes: Extracellular organelles important in intercellular communication. Journal of Proteomics, 73, 1907–1920. 20601276 10.1016/j.jprot.2010.06.006

[mbt214274-bib-0016] Moradi‐Chaleshtori, M. , Bandehpour, M. , Heidari, N. , Mohammadi‐Yeganeh, S. & Hashemi, S.M. (2021) Exosome‐mediated miR‐33 transfer induces M1 polarization in mouse macrophages and exerts antitumor effect in 4T1 breast cancer cell line. International Immunopharmacology, 90, 107198. 33249048 10.1016/j.intimp.2020.107198

[mbt214274-bib-0017] Ranjbar, R. , Sharifimoghadam, S. , Saeidi, E. , Jonaidi, N. & Sheikhshahrokh, A. (2016) Induction of protective immune responses in mice by double DNA vaccine encoding of *Brucella melitensis* omp31 and *Escherichia coli* Eae genes. Tropical Journal of Pharmaceutical Research, 15, 2077–2083.

[mbt214274-bib-0018] Shah, V.O. , Ferguson, J. & Hunsaker, L.A. (2011) Cardiac glycosides inhibit LPS‐induced activation of pro‐inflammatory cytokines in whole blood through an NF‐κB‐dependent mechanism. International Journal of Applied Research in Natural Products, 4, 11–19.

[mbt214274-bib-0019] Ti, D. , Hao, H. , Tong, C. , Liu, J. & Dong, L. (2015) LPS‐preconditioned mesenchymal stromal cells modify macrophage polarization for resolution of chronic inflammation via exosome‐shuttled let‐7b. Journal of Translational Medicine, 13, 1–14. 26386558 10.1186/s12967-015-0642-6 PMC4575470

[mbt214274-bib-0020] Verreck, F.A.W. , De Boer, T. , Langenberg, D.M.L. , Hoeve, M.A. , Kramer, M. , Vaisberg, E. et al. (2004) Human IL‐23‐producing type 1 macrophages promote but IL‐10‐producing type 2 macrophages subvert immunity to (myco)bacteria. Proceedings of the National Academy of Sciences of the United States of America, 101, 4560–4565. 15070757 10.1073/pnas.0400983101 PMC384786

[mbt214274-bib-0021] Wang, T. , Nasser, M.I. , Shen, J. , Qu, S. , He, Q. & Zhao, M. (2019) Functions of exosomes in the triangular relationship between the tumor, inflammation, and immunity in the tumor microenvironment. Journal of Immunology Research, 2019, 10. 10.1155/2019/4197829 PMC6701352 31467934

[mbt214274-bib-0022] Wang, Y.C. , He, F. , Feng, F. , Liu, X.W. , Dong, G.Y. , Qin, H.Y. et al. (2010) Notch signaling determines the M1 versus M2 polarization of macrophages in antitumor immune responses. Cancer Research, 70, 4840–4849. 20501839 10.1158/0008-5472.CAN-10-0269

[mbt214274-bib-0023] Xu, X. , Xu, J. , Wu, J. , Hu, Y. , Han, Y. , Gu, Y. et al. (2021) Erratum: Phosphorylation‐mediated IFN‐γ R2 membrane translocation is required to activate macrophage innate response. Cell, 184, 1393–1394. 33667370 10.1016/j.cell.2020.02.037

[mbt214274-bib-0024] Yi, J. , Wang, Y. , Zhang, H. , Deng, X. , Xi, J. , Li, H. et al. (2021) Interferon‐inducible transmembrane protein 3‐containing exosome as a new carrier for the cell‐to‐cell transmission of anti‐*Brucella* activity. Frontiers in Veterinary Science, 8, 1–11. 10.3389/fvets.2021.642968 PMC8010673 33816587

[mbt214274-bib-0025] Zhang, F. , Li, Z. & Jia, B. (2019) The immunogenicity of OMP31 peptides and its protection against *Brucella melitensis* infection in mice. Scientific Reports, 9, 3512. 30837598 10.1038/s41598-019-40084-w PMC6401381

[mbt214274-bib-0026] Zhao, X. , Wu, D. , Ma, X. , Wang, J. , Hou, W. & Zhang, W. (2020) Exosomes as drug carriers for cancer therapy and challenges regarding exosome uptake. Biomedicine & Pharmacotherapy, 128, 110237. 32470747 10.1016/j.biopha.2020.110237

